# Cavernous brain malformations and their relation to black blood MRI in respect to vessel wall contrast enhancement

**DOI:** 10.1186/s41016-018-0116-9

**Published:** 2018-05-08

**Authors:** Athanasios K. Petridis, Marian P. Suresh, Jan F. Cornelius, Richard Bostelmann, Maxine Dibué-Adjei, Lan Li, Marcel A. Kamp, Hans Jakob Steiger, Bernd Turowski, Rebecca May

**Affiliations:** 1 Department of Neurosurgery, Heinrich Heine University Düsseldorf, Moorenstr 5, 40225 Düsseldorf, Germany; 2 Diagnostic and Interventional Neuroradiology, Heinrich Heine University Düsseldorf, Düsseldorf, Germany; 3 LivaNova Deutschland (a LivaNova PLC-owned subsidiary), Lindberghstr 25, 80939 Munich, Germany

**Keywords:** Cavernous malformations, Inflammation, Black blood MRI

## Abstract

**Background:**

Inflammatory responses are implicated as crucial patho-mechanisms of vascular brain malformations. Inflammation is suggested to be a key contributor to aneurysm rupture; however it is unclear whether inflammation contributes similarly to bleeding of cerebral cavernous malformations (CCMs). Black blood MRI is a sequence which identifies inflammation in blood vessel walls and in the present study is used to detect inflammatory response in CCMs.

**Methods:**

Fifteen patients with 17 CCMs treated in our department in 2017 were retrospectively analysed. All patients received black blood MRIs and the results were analysed in correlation with, size and bleeding of CCMs.

**Results:**

Size and bleeding status of CCMs did not correlate with contrast enhancement in the CCM wall. One of 3 patients with bleeding displayed contrast enhancement in black blood MRI, whereas the others had non enhancing lesions. Because of the small number of cases a statistical analysis was not performed.

**Conclusion:**

In this limited cohort, inflammatory reactions in CCMs could not be detected by black blood MRI suggesting that the level of inflammation is minimal in these lesions and those different patho-mechanisms play a more important role in the rupture of CCMs.

## Background

Inflammatory responses are suggested to play an important role in intracranial aneurysm behaviour, growth and rupture [1, 2]. The high resolution magnetic resonance imaging technique known as black blood MRI (bbMRI) can be used to identify inflammatory processes in the aneurysm wall and therefore its use in predicting which aneurysms are prone to rupture is of interest [3, 4]. The bbMRI is used primarily in extracranial steno occlusive carotid disorders but is finding increasing use in intracranial aneurysms and arterial dissections [3–6].

A number of studies demonstrate that inflammatory processes may lead to growth and bleeding of cerebral cavernous malformations (CCM). Macrophages and B cells surrounding leaky vessels in the CCM is well documented, however whether this is simply a reaction to bleeding or represents an inflammatory response leading to further proliferation of the CCM is not as clear [7]. With an incidence of around 0.5% and unclear proliferative behaviour it would be interesting to predict which CCMs proliferate and are at risk of bleeding [8]. Shi et al. could show the presence of oligo clonal patterns of IgG which are produced from B cells and plasma cells within the CCM leading to an inflammatory response and probably to instability of the CCM walls [7].

In this present retrospective analysis we investigated the possibility of identifying inflammatory processes in CCMs with bbMRI by evaluating potential correlation between CCM growth, bleeding and contrast enhancement.

## Methods

From January 2017 to May 2017 patients with CCMs presenting in our department received a bbMRI sequence in addition to routine imaging. We then retrospectively investigated the MRIs of all patients with CCMs treated in our department. The patients presented for follow up after 2 years in our outpatient facilities where they received an MRI or in case of CCM bleeding in the emergency room. We perform a follow up for non-bleeding CCMs by MRI every 2 years. Analysis of bbMRI’s was carried out by one radiologist and contrast enhancement in the CCM and around the lesion was quantified by a second radiologist CCM size was defined as the longest diameter in mm.

Cranial MRI was performed on a 3 T MR scanner (Magnetom Skyra, Siemens, Erlangen) with a 20 channel head coil. The protocol included a 3 D T1 space sequence with fat saturation (SPAIR) and blood suppression (field of view 179 × 230, repetition time 693 ms, echo time 18 ms, matrix 256 × 256, spatial resolution 0.9 x .09 × 0.9 mm) before and after administration of gadolinium (0,2 ml/kg/BW, maximum 20 ml; ProHance, Bracco Imaging, Germany). Total scan time was 7:55 min.

## Results

From January 2017 to May 2017 *n* = 15 patients with CCMs received a bbMRI for detection of vessel wall inflammation in our department. Nine patients were female and 6 male. The mean age was 53 years (28–84 y.o., SD: ±17 years). Two patients had multiple CCMs but no known family members with the disease. Three patients presented in our emergency room with bleeding. One of these patients had an occipital CCM and was treated surgically; the other 2 had brainstem CCMs and were treated conservatively with subsequent improvement of symptoms and discharge after 7 days. All 3 patients with bleeding had clinical symptoms related to the bleeding localisation: diplopia or other or visual disturbances as well as a mild hemipalsy or gait disturbances in the case of the brainstem CCMs. Eight patients had known CCMs and came for follow up (mean follow up time 3.4 years, 1 month-20 years, follow up intervals: 2 years). The CCMs (*N* = 17) had a mean size of 11 mm (SD ± 7 mm, 3–27 mm).

Two malformations were localized in the occipital lobe, 2 parietal, 1 frontal, 1 temporal, 1 parahippocampal, 4 cerebellar, 1 striatal and 5 in the brainstem.

Five cavernomas were classified as Zabramski I, 3 Zabramski II, 6 Zabramski III and only 1 Zabramski IV. The other lesions were not distinctly classifiable [9].

Only one CCM showed contrast enhancement in the bbMRI: a bleeding brainstem cavernous malformation (22 mm size) in a patient with two lesions. The second lesion did not bleed and was not contrast enhancing. The patient was treated conservatively and after 2 months his symptoms of mild gait ataxia and double vision were resolved completely. In the two months follow up the previously contrast enhancing CCM showed nearly complete resolution of blood and no contrast enhancement. All other lesions did not display contrast enhancement irrespective of size, bleeding, multiplicity, gender, or age. One lesion was accompanied by a developmental venous anomaly and some contrast enhancement was localized in the transition zone between CCM and dural venous anomaly. None of the CCMs in the follow up showed a proliferation, therefore we could not study contrast enhancement and proliferation. Statistical analysis would not make any sense in these findings.

Table 1 summarizes some of the characteristics of the CCMs in the 15 studied patients.

**Table 1:** Characteristics of cavernomas in the 15 studied patients

• *Patient*	• *Age (years)*	• *Gender*	• *Cavernom Size (mm)*	• *Localisation*	• *Bleeding*	• *Growth*	• *Multiple*	• *Black Blood Contrast enhancement*
1	39	f	14	occipital	yes	n/a	no	no
2	39	f	10	pedunculus cerebri	no	no	no	No
3	58	f	7	parietal	no	no	no	No
4	44	f	7	parietal	no	no	no	No
5	84	f	17	brainstem	no	no	no	No
6	28	f	27	Pons	1 month ago	no	yes	No
7	54	m	22 and 9	Mesencephalon and temporoccipital	yes	no	yes	yes
8	71	f	13 and 3	cerebellar hemisphere and parahippocampal	no	no	no	No
9	28	m	9	cerebellar hemisphere	no		no	No
10	61	f	5	cerbellar hemisphere	no		no	No
11	63	m	7	cerebellar hemisphere	no	no	no	yes
12	55	m	6	Mesencephalon	5 years ago	no	no	No
13	73	f	7	frontoparietal	yes	n/a	no	No
14	39	f	14	occipital	no	n/a	no	No
15	39	f	10	pedunculus cerebri	no	no	no	No

## Discussion

**Fig. 1:**
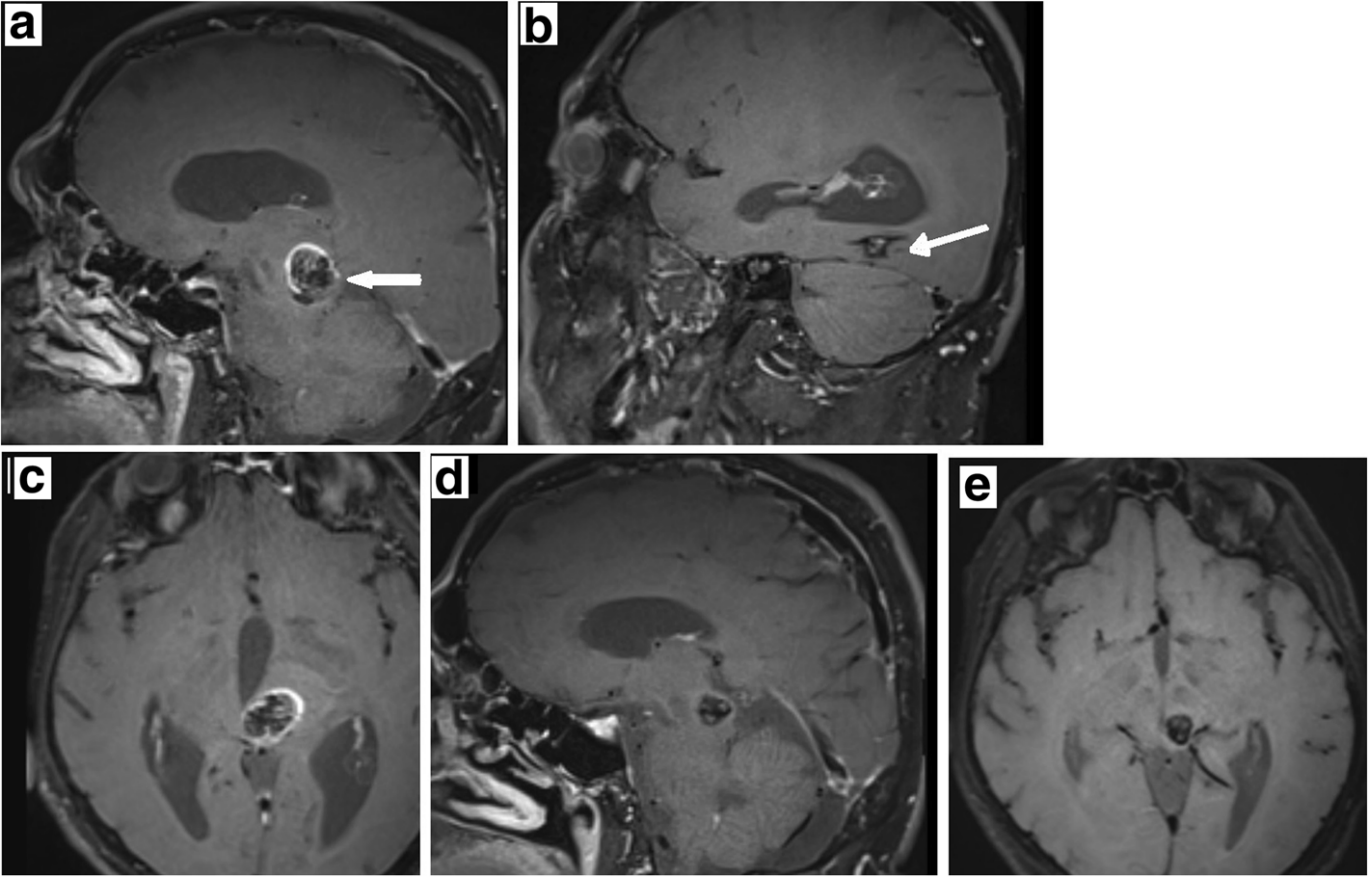
Intracerebral bleeding of a mesencephalic CCM, **a** Sagittal plane of a black blood MRI showing contrast enhancement of the mesencephalic CCM (arrow) (ruptured) (left). **b** Non bleeding second CCM localised in the occipital lobe (right) without contrast enhancement (arrow:CCM). **c** Same as in 1A. Black blood transverse plane of the mesencephalic CCM. **d** Sagittal section. Follow up black blood MRI 2 months after bleeding. The CCM blood is completely resorbed and no contrast enhancement can be seen. **e** Transverse section of D

Inflammatory processes are present in CCMs which are shown by the presence of macrophages and B cells in surgical specimens of such malformations [7]. However when and to which degree inflammation plays a crucial part in CCMs has yet to be elucidated. Shi et al. show production of oligo clonal IgGs in CCM lesions indicating inflammatory processes, possibly leading to proliferation of CCMs [7]. However, these processes may be so slow and minimal that they do not lead to contrast enhancement in the bbMRI explaining the failure of predicting CCM proliferation by bbMRI imaging. In all our follow up cases CCMs were stable in size which clearly indicates a very slow proliferation rate of those lesions. Even in the case we present in this study there is an immediate inflammatory response after bleeding, which is probably attributable to a macrophage surge, but seems to be absent two months after bleeding (at least in MRI imaging, Fig. 1). Hence proliferation of CCMs may represent a process that is not rapid or robust in dimension and therefore not detectable by bbMRI.

This stands in contrast to aneurysm growth which appears to be triggered by a fast inflammation seen in bbMRI’s suggesting differences in growth behaviour and molecular mechanisms in the two pathologies.

The present analysis is limited primarily by the small amount of patients, which prevents drawing ultimate conclusions from the three ruptured CCMs. Furthermore the analysis is limited by its retrospective nature and the lack of histological analysis of the CCMs. However, despite these limitations we give initial insight into the use of bbMRI for prediction of CCM rupture reporting that bbMRI did not identify unstable CCMs in our patients. In 15 patients with 17 CCM contrast enhancement did not correlate with size or stage of the CCM. Since bbMRI depicts inflammation in the vessel walls and in aneurysm walls one could conclude that inflammation in the CCM walls is minimal and does not seem to play a crucial role in rupture since only 1 of 3 cases in our analysis showed a contrast enhancement in bbMRI. To our knowledge this is the first study investigating bbMRI for identification of unstable CCMs and further studies with larger sample size will be necessary to investigate potential non-invasive imaging methods capable of quantifying CCM instability.

The limitations of the study do not allow a further conclusion since the study is retrospective and we have no histological analysis of the CCMs. On the other hand most cavernomas show histologically no inflammation and this is correlating with the non-enhancing bbMRIs.

## Conclusion

Even that the data present negative results it is important to show the limits of the new imaging (black blood MRI) in different vascular pathologies of the CNS. Different than in intracranial aneurysms and AVM contrast enhancement in the walls of cavernomas seems to be irrelevant of their maturation stage.

## References

[1] Peng H, Yang Q, Wang DD, Guan SC, Zhang HQ (2016). Wall enhancement on high-resolution magnetic resonance imaging may predict an unsteady state of an intracranial saccular aneurysm. Neuroradiology.

[2] Thompson BG, Brown RD, Amin-Hanjani S, Broderick JP, Cockroft KM, Connoly ES (2015). Prevention. Guidelines for the management of patients with unruptured intracranial aneurysms: a guideline for healthcare professionals from the American Heart Association/American stroke association. Stroke; J Cereb Circ.

[3] Coppenrath E, Lenz O, Sommer N, Lummel N, Linn J, Treitl K (2017). Clinical significance of intraluminal contrast enhancement in patients with spontaneous cervical artery dissection: a black-blood MRI study.

[4] Edelman RR, Chien D, Kim D (1991). Fast selective black blood MR imaging. Radiology.

[5] Edelmann R, Mattle HP, Wallner R (1990). Extracranial carotid arteries: evaluation with “black blood” MR angiography. Radiology.

[6] Melhem ER, Jara H, Yucel EK (1997). Black blood MR angiography using multislab three-dimensional T1-weighted turbo spin-echo technique: imaging of intracranial circulation. AJR.

[7] Shi C, Shenkar R, Batjer H, Check IJ, Awad IA (2007). Oligo clonal immune response in cerebral cavernous malformations. J Neurosurg.

[8] Del Curling O Jr, Kelly DL Jr, Elster AD, Craven TE. An analysis of the natural history of cavernous angiomas. J Neurosurg 1991; 75: 702–708. 10.3171/jns.1991.75.5.0702 1919691

[9] Zabramski JM, Wascher TM, Spetzler RF, Johnson B, Golfinos J, Drayer BP (1994). The natural history of familial cavernous malformations: results of an ongoing study. J Neurosurg.

